# Silica-supported Pt(0) single-atom catalyst chemisorbing hydrogen but converting to Pt(iv) by ambient oxygen

**DOI:** 10.1039/d6ra00018e

**Published:** 2026-03-02

**Authors:** Gwang-Jin Na, Jongha Hwang, Ryong Ryoo

**Affiliations:** a Department of Energy Engineering, Korea Institute of Energy Technology (KENTECH) Naju Jeonnam 58330 Korea rryoo@kentech.ac.kr

## Abstract

We confirmed the stabilization of Pt(0) single atoms on porous silica using electron microscopy and X-ray absorption. The Pt/silica exhibited strong chemisorption of 2.0H/Pt, but no catalytic activity was observed for olefin hydrogenation. The Pt(0) state was changed to Pt(iv) by ambient O_2_, which led to doubt about oxidation catalytic abilities as Pt(0).

## Abstract 2

Pt(0) single atoms on porous silica show strong hydrogen chemisorption (2.0H/Pt) but no olefin hydrogenation activity, and are readily oxidized to Pt(iv) upon exposure to oxygen.

## Graphical abstract

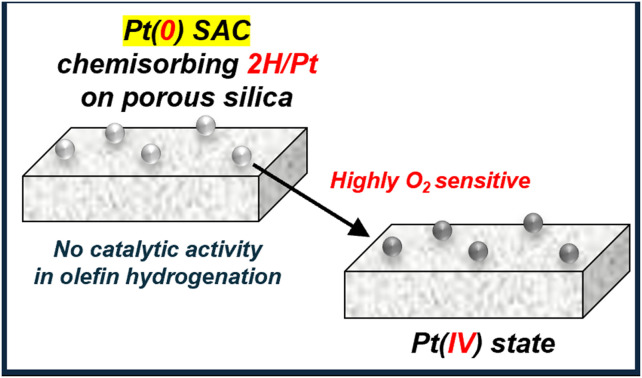

Platinum is one of the most important catalysts currently used in various chemical processes, such as the removal of pollutants from automobile exhaust gas,^1^ manufacturing of hydrogen by electrolysis,^2,3^ generation of electricity using fuel cells^4^ and the production of commodity raw materials *via* petrochemical processing.^5,6^ In conventional applications, the Pt catalyst is supported on porous materials with high specific surface areas, such as carbon, silica (SiO_2_), alumina (Al_2_O_3_), titania (TiO_2_), and ceria (CeO_2_). The role of the supports is to disperse Pt into tiny nanoparticles so that a high fraction of Pt atoms can be exposed to the surface for catalytic reactions. The surface-exposed fraction of the metal atoms is called ‘catalyst dispersion’, which approaches 100% as the diameter decreases to less than 1 nm. The catalysts, consisting of only a few Pt atoms, have attracted specific attention in heterogeneous catalysis for several decades due to the discretization of electronic energy levels from continuous bands of bulk metal. In recent years, this cutting-edge interest has been shifting to the complete dispersion of Pt metal catalysts into single atoms.

The single-atom catalyst (SAC) is emerging as a new, exciting frontier in catalysis.^7,8^ The single-atom nature, lacking metal–metal bonds with neighboring atoms, is also considered to provide distinct coordination environments for reactant molecules compared with those around bulk surface atoms. The SAC may resemble a homogeneous catalyst anchored to the surface of a porous solid. Recent investigations on SAC have reported superior selectivity and conversion rates compared with those of bulk metal catalysts or nanoparticles. These studies were often focused on SACs supported on porous carbons,^9,10^ CeO_2_,^11,12^ and other transition metal oxides, which could exert strong metal–support interactions (SMSI).^13–15^ Regarding the carbon-supported SACs, there is a general agreement that the catalyst is metal ion-stabilized through coordination bonding with carbon frameworks.^9,10^ In the case of the metal oxide support, the catalytic atoms can be stabilized through bonding with oxygen on the oxide surfaces.^13–15^ The catalytic function in these SAC systems could predominantly arise from catalyst atoms existing in an ionic state or oxide. Particularly, in the case of Pt/CeO_2_, the electron transfer from Pt atoms to ceria alters the electronic nature of the catalyst atoms substantially.^11,12,16^ This phenomenon raises questions about whether the catalysis can truly be attributed to the SAC of metals in the zero oxidation state.

To obtain single Pt atoms in the metal-like zero oxidation state, *i.e*., the Pt(0) SAC, it is important to disperse Pt on the surface of an inert support that does not have SMSI. Porous silica is ideal as an inert support, but the lack of SMSI makes it difficult for the silica surface to stabilize singly dispersed Pt(0) atoms without agglomeration. The dilemma between the inertness requirement and the prevention of Pt agglomeration has been a challenge to date to study the nature of the Pt(0) SAC. In this regard, we adopted an approach utilizing the entropic dispersion effect while decreasing the Pt loading (*i.e.*, Pt content in wt%) on SiO_2_ to extremely low values. This entropic effect at ultralow Pt loading was monitored by hydrogen chemisorption, for which a special volumetric adsorption apparatus was built. Images of the dispersed Pt atoms were obtained by atomic-resolution scanning transmission electron microscopy (STEM). Based on this approach, appropriate experimental conditions to disperse the Pt(0) SAC on SiO_2_ were established. The Pt oxidation state, thus obtained, was probed by X-ray absorption near-edge structure (XANES) under H_2_ and after exposure to air. Extended X-ray absorption fine structure (EXAFS) was used to confirm the single atomic Pt environment. Above all, we paid particular attention to whether the Pt(0) SAC obtained in this manner could chemisorb hydrogen and therefore catalyze the hydrogenation of olefins, such as *n*-hexene (*n*-C_6_), propylene (C_3_), and ethylene (C_2_).

The dispersion of Pt/SiO_2_ was performed by following an incipient wetness-impregnation process, which was conventionally used to achieve high dispersion of Pt catalysts on porous solids (see SI for sample preparation). In this process, a preset amount of aqueous Pt(NH_3_)_4_(NO_3_)_2_ solution was impregnated into porous silica to have a desired Pt loading in the range of 0.01–1.0 wt% Pt. The Pt precursor was converted to PtO_2_ while heating in O_2_. The Pt(iv) oxide was then converted to Pt(0) by heating in H_2_. The Pt(0) could be dispersed into single atoms or aggregated, depending on the Pt loading and the treatment temperatures in O_2_ and H_2_. The resultant effects on Pt dispersion were analyzed using H chemisorption, STEM, and XANES.

The results indicated that the dispersed state of Pt atoms, *i.e*., either single atoms or agglomeration into nanoparticles, depended critically on the treatment temperatures in O_2_ and H_2_, as well as Pt loadings. Above all, the precursor had to be impregnated to give an ultralow concentration of Pt, so that Pt(NH_3_)_4_(NO_3_)_2_ could be well dispersed in a single-molecular manner. The single-molecularly dispersed Pt precursor could be converted to PtO_2_ by heating to above 290 °C in O_2_, similar to the cases of Pt/zeolite and Pt/alumina.^17–25^ The dispersion into single PtO_2_ molecules or agglomeration on the present SiO_2_ depended critically on the O_2_ temperature. After heating in O_2_ at around 290–300 °C, the resultant PtO_2_ was confirmed to be atomically dispersed. High-temperature heating above 350 °C resulted in Pt agglomeration, which could be attributed to the volatility of the metal oxide.^26^ The resultant PtO_2_/SiO_2_, in a single molecular dispersion, was readily converted into the Pt(0) state by heating to 200 °C in H_2_ [see the H_2_-temperature-programmed reduction (TPR) profiles in SI and Fig. S1]. Here, limiting the reduction temperature to below 200 °C was critical to maintain the single atomic Pt(0) dispersion on SiO_2_. H_2_ treatment at an excessively high temperature led to thermal agglomeration of the resultant Pt(0) atoms. Considering all these factors, we optimized the Pt SAC-dispersing conditions as follows: Pt loading on SiO_2_ ≤ 0.02 wt% (determined by ICP-AES), O_2_ treatment at 300 °C, and a subsequent H_2_ treatment at 200 °C.

The entropic dilution effect for the Pt/SiO_2_ dispersion can be checked with H chemisorption, as shown in Fig. 1. The H/Pt ratio on the *y*-axis represents the atomic ratio between chemisorbed H atoms and total Pt atoms, determined by extrapolating volumetrically measured H adsorption isotherms to zero pressure. Normally, such a volumetric adsorption measurement is difficult to apply for Pt loadings below 0.5 wt% since the extraction of a small H/Pt ratio from the large amount of physisorption on SiO_2_ may not be accurate in the extrapolation process. To extend the application range to 0.01–0.02 wt% Pt, we utilized a laboratory-built borosilicate glass adsorption apparatus. In this apparatus, adsorption quantities were measured by monitoring pressure changes with a Baratron capacitance manometer. However, in this gauge, the capacitance diaphragm deflects depending on the dosing gas pressure. Even a slight deflection can cause a significant error in H chemisorption when the Pt loading is lower than 0.5 wt%. Hence, the deflection effect was corrected in the present work on ultralow Pt loadings (see SI for H chemisorption measurements).

**Fig. 1:**
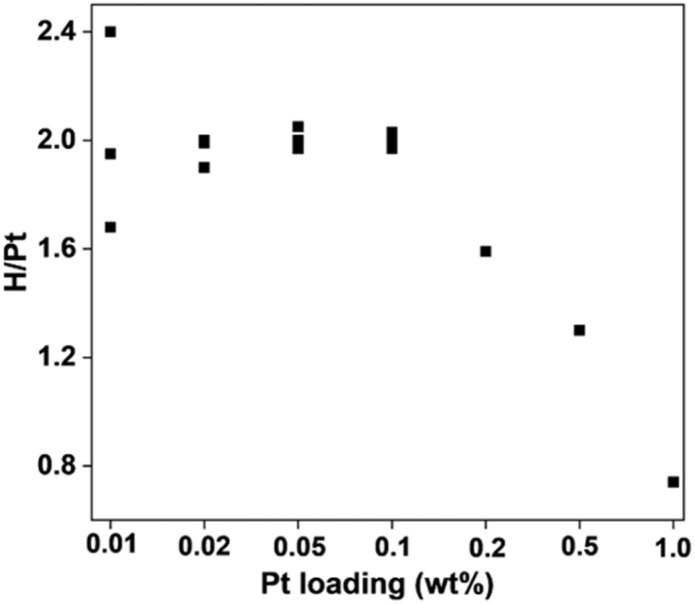
Hydrogen chemisorption expressed as H/Pt ratios for various Pt loadings on SiO_2_.

As Fig. 1 shows, the H/Pt ratio increased from 0.7 to 2.0 as the Pt loading decreased from 1.0 to 0.1 wt% on SiO_2_. Upon further decrease, the H/Pt ratio remained around 2.0 despite a 10-fold reduction in the Pt concentration (the corresponding H adsorption isotherms are shown in Fig. S2). The plateaued values over 0.1–0.01 wt% Pt suggest that 2.0H/Pt could be the maximum limit for Pt. Assuming 100% dispersion at this plateau, all the Pt atoms would be surface-exposed and chemisorb two hydrogen atoms each. Three possibilities may be considered for such dispersed Pt: monolayered rafts, nanoparticles consisting of a few atoms, and single atomic dispersion.

The high-angle annular dark-field (HAADF) STEM images of the 0.02 wt% Pt/SiO_2_ sample revealed single-atomically dispersed Pt atoms (Fig. 2 and S3a for the corresponding high-contrast HAADF-STEM image), which was further confirmed by the corresponding line-scanning analysis (Fig. S3b). No significant amounts of Pt agglomerates were detected in the 0.02 wt% Pt/SiO_2_ (Fig. S4). Increasing the Pt loading to 0.05 and 0.1 wt% caused conspicuous Pt agglomeration into small clusters (5–15 atoms/cluster) (Fig. S5). Here, all HAADF-STEM images were obtained after stabilization of the dispersed Pt atoms *via* passivation under a 0.1% O_2_/N_2_ flow, preventing Pt agglomeration upon sudden exposure to air. The passivated Pt/SiO_2_ samples could be safely re-reduced in flowing H_2_ at 200 °C after degassing at room temperature (RT). The reduction at higher temperatures caused the agglomeration of Pt atoms (Fig. S6).

**Fig. 2:**
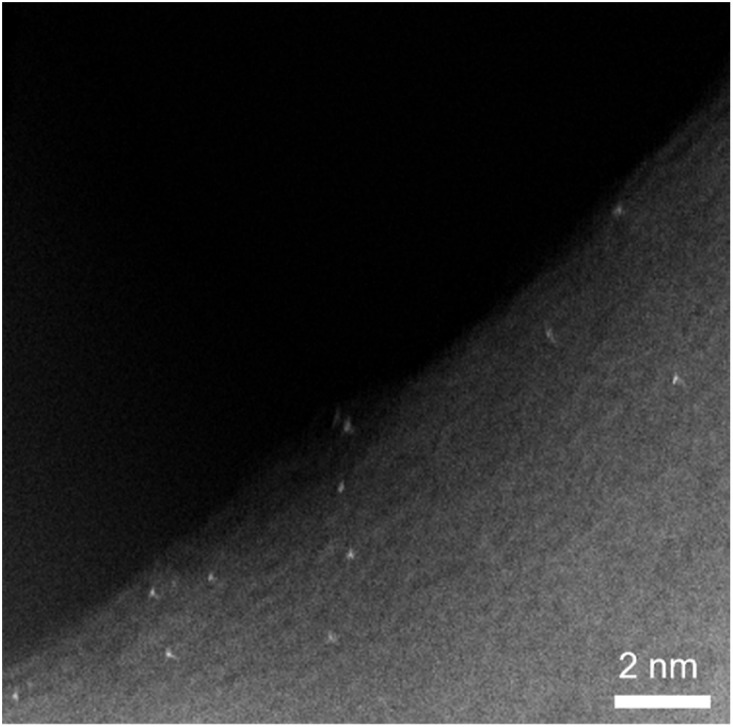
HAADF-STEM image of 0.02 wt% Pt/SiO_2_ showing single Pt atoms.

The Pt oxidation state was monitored by obtaining PtL_3_ edge XANES spectra in each preparation step of the single atomically dispersed 0.02 wt% Pt/SiO_2_. The three PtL_3_ XANES data shown in Fig. 3a were taken in three representative steps of the sample preparation, following the precursor impregnation. The XANES data in the dashed line were obtained under O_2_ at RT after treatment in O_2_ at 300 °C. The XANES in solid lines was taken in H_2_ after subsequent heating in H_2_ at 200 °C. This H_2_-treated sample was the single-atomically dispersed 0.02 wt% Pt/SiO_2_ shown in the HAADF-STEM image in Fig. 2. The third XANES presented in dotted lines in Fig. 3a was taken after passivation of the single atomic 0.02 wt% Pt/SiO_2_ in 0.1% O_2_/N_2_ at RT, which was almost identical to the O_2_-treated stage.

**Fig. 3:**
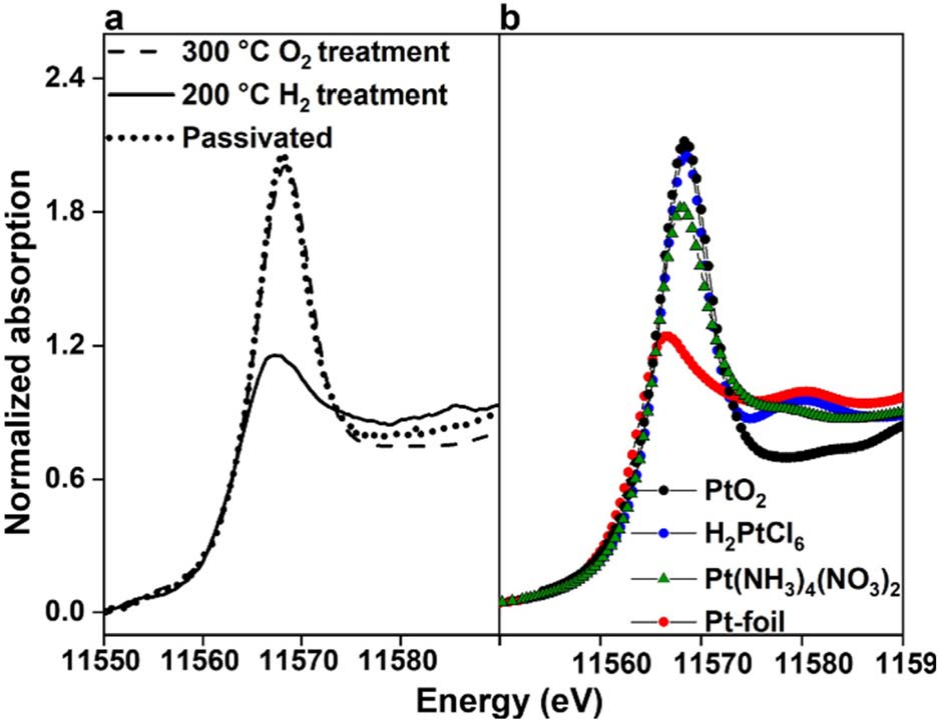
Normalized XANES data obtained at the PtL_3_ edge at RT. (a) 0.02 wt% Pt/SiO_2_ at different steps of preparation: (---) exposed to air after the O_2_ treatment of Pt(NH_3_)_4_(NO_3_)_2_/SiO_2_ at 300 °C, (—) under H_2_ gas after the H_2_ treatment at 200 °C, following the O_2_ treatment at 300 °C, and (···) passivated after the H_2_ treatment with 0.1% O_2_/N_2_ at 200 °C. (b) Pt reference samples: Pt foil, Pt(NH_3_)_4_(NO_3_)_2_, H_2_PtCl_6_, and PtO_2_, representing the oxidation states of Pt(0), Pt(ii), Pt(iv) and Pt(iv), respectively.

All the PtL_3_ XANES spectra presented in Fig. 3a exhibited a sharp, intense peak over 11 564–11 574 eV. This peak, continuously rising from the absorption edge at 11 564 eV, is called ‘white line’. The white line is related to electronic transitions from the core 2p_3/2_ level to empty 5d_3/2_, 5d_5/2_ and 6s valence states. Hence, the white line intensity can be used to probe the unoccupied electron densities in the valence atomic orbitals. In particular, Pt oxidation states can be determined in comparison to the reference compounds of Pt foil, Pt(NH_3_)_4_(NO_3_)_2_, H_2_PtCl_6_, and PtO_2_, as shown in Fig. 3b. On this ground, the XANES result presented in the dashed line in Fig. 3a may be interpreted by the change of the Pt oxidation state to Pt(iv) from the Pt(ii) state of the Pt(NH_3_)_4_(NO_3_)_2_ precursor when treated in O_2_ at 300 °C (Fig. S7a and b). This is probably due to the formation of a single molecularly dispersed PtO_2_ species on the silica surface. Upon subsequent treatment in H_2_ at 200 °C, the oxidation state appeared to be lowered to Pt(0), as in the metallic Pt foil. The reduced Pt(0) species is considered to remain atomically dispersed on the silica surface, as judged by this XANES analysis in combination with HAADF-STEM images. The analysis results indicated that the single atomic Pt(0) was stable under H_2_, but immediately converted to PtO_2_ when exposed to air at RT. Once passivated with 0.1% O_2_/N_2_ at RT, the stabilized PtO_2_ species could be safely re-reduced back to the single atomic Pt(0) state. The re-reduced 0.02 wt% Pt(0)/SiO_2_ exhibited identical properties of H chemisorption and XANES, as compared to the freshly prepared sample. The EXAFS analysis also indicated no detectable Pt–Pt coordination (Fig. S7c).

Catalytic properties of 0.02 wt% Pt(0)/SiO_2_ SAC for *n*-C_6_ hydrogenation are presented in Fig. 4, in comparison to the catalytic activity of agglomerated Pt atoms (*i.e*., nanoparticles). As shown in the results, the nanoparticle catalyst exhibited a steady conversion of *n*-C_6_ at −20 °C during the entire 60 min measurement time. In contrast, the SAC exhibited no conversion. Upon increasing the reaction temperature to 0 °C or 20 °C (Fig. S8), the SAC maintained zero conversion or suddenly jumped into high conversions during the reaction time (about 5% conversion at 15 min in the case of 0 °C reaction shown in Fig. 4). We attribute the sudden change to the result of Pt agglomeration by a thermal runaway (STEM and EXAFS in Fig. S9), which could be initiated due to the heat of reaction occurring at surfaces of Pt clusters that might exist sparsely among single Pt atoms. Such thermal runaways immediately occurred at the start of the reaction or during measurements, depending on reaction temperatures, olefin species and concentrations. To avoid the problem, the reaction temperature and the olefin feeding rate had to be set as low as possible. When the hydrogenation was measured without the thermal runaway effect, all the C_2_, C_3_ and *n*-C_6_ olefins exhibited zero conversion under the present conditions (Table S1). As the result indicates, the Pt(0) SAC supported on SiO_2_ is catalytically inactive for the hydrogenation reaction. However, catalytic activity may be markedly different when Pt atoms are dispersed on other metal surfaces (*e.g.*, Au) or reducible metal oxides (*e.g.*, Fe_2_O_3_ and CeO_2_).^11–13^ Pt atoms, coordinated to the surface metal atoms, may be catalytically active, similar to the result in Pt clusters.

**Fig. 4:**
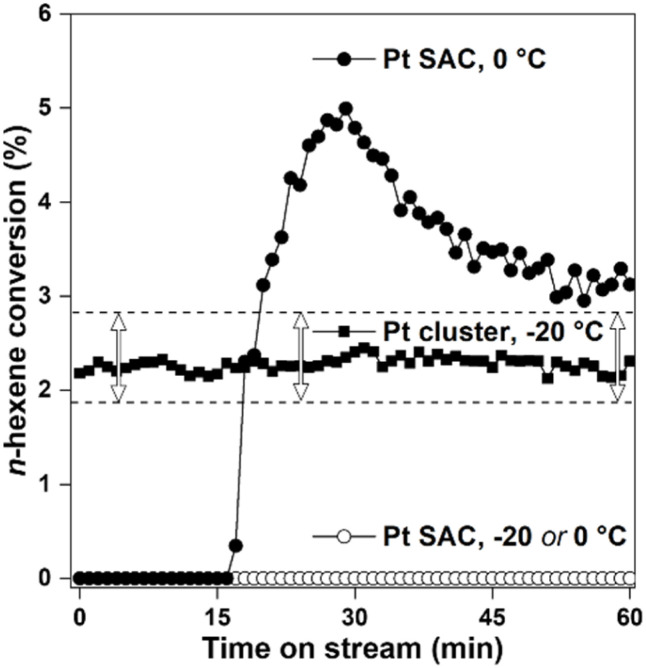
Hydrogenation of *n*-hexene with 20 mg of the catalyst measured at the reactant feeding rate of WHSV = 1700 h^−1^ and H_2_/*n*-hexene = 164. ‘SAC’ denotes the 0.02 wt% Pt/SiO_2_ single-atom catalyst shown in Fig. 2, which was prepared by heating at 200 °C in H_2_. ‘Pt cluster’ refers to the agglomeration of Pt atoms into nanoparticles *via* heating at 400 °C in H_2_ (Fig. S6b). Numbers followed by °C indicate the reaction temperatures. Arrows indicate the error ranges confirmed with 3 different runs.

To ensure that the formation of the Pt(0) SAC on silica was free from impurity-related effects, we prepared another series of 0.02 wt% Pt/SiO_2_ catalysts using high-purity silica, which was synthesized in the form of hierarchically meso-microporous MFI zeolite developed in our laboratory (Fig. S10).^27^ The ICP analysis indicated that the lab-made zeolitic silica contained much lower levels of impurities (mainly Na^+^) than those present in the Davisil silica used in the present work (Table S2). Despite the conspicuous difference in impurity levels and silica structures, the 0.02 wt% Pt loading on the two silica samples showed identical results in H chemisorption, and exhibited no catalytic activity differences in hydrogenation reactions (Fig. S11). In this manner, we confirmed that the unique characteristics of the Pt(0) SAC were not influenced by impurity effects.

The present work shows that a conventional impregnation method can readily be applied to prepare a cutting-edge Pt/SiO_2_ material in which the Pt element truly exists in the single-atomically dispersed Pt(0) state. The Pt(0)/SiO_2_ SAC exhibited strong chemisorption of 2.0H/Pt, but still, the Pt atoms without Pt–Pt ensembles could not perform the catalytic hydrogenation of C_2_, C_3_ and *n*-C_6_ olefins. Another noteworthy point is that the Pt(0) SAC was sensitive to O_2_, so that the Pt was immediately oxidized into a Pt(iv) state even by a brief contact with air at room temperature. The O_2_ sensitivity suggests that the Pt(0) SAC would not be a catalytically active site for oxidation reactions under O_2_, but probably, the catalytic state could be Pt(iv). These findings could further motivate future mechanistic studies to explore the catalytic relevance of oxidized Pt species, including Pt(iv), in reactions under oxidative environments.

## Conflicts of interest

There are no conflicts to declare.

## Supplementary Material

RA-016-D6RA00018E-s001

## Data Availability

The data supporting this article have been included as part of the supplementary information (SI). Supplementary information is available. See DOI: https://doi.org/10.1039/d6ra00018e.

## References

[cit1] Yam V. W. W. (2010). Nat. Chem..

[cit2] Zeng L., Zhao Z., Lv F., Xia Z., Lu S.-Y., Li J., Sun K., Wang K., Sun Y., Huang Q., Chen Y., Zhang Q., Gu L., Lu G., Guo S. (2022). Nat. Commun..

[cit3] Zeng H., Chen Z., Jiang Q., Zhong Q., Ji Y., Chen Y., Li J., Liu C., Zhang R., Tang J., Xiong X., Zhang Z., Chen Z., Dai Y., Li C., Chen Y., Zhao D., Li X., Zheng T., Xu X., Xia C. (2025). Nat. Commun..

[cit4] Ren X., Wang Y., Liu A., Zhang Z., Lv Q., Liu B. (2020). J. Mater. Chem. A.

[cit5] Yu W. (2012). Chem. Rev..

[cit6] Ryoo R., Kim J., Jo C., Han S. W., Kim J.-C., Park H., Han J., Shin H. S., Shin J. W. (2020). Nature.

[cit7] Wang A., Li J., Zhang T. (2018). Nat. Rev. Chem..

[cit8] Kment Š., Bakandritsos A., Tantis I., Kmentová H., Zuo Y., Henrotte O., Naldoni A., Otyepka M., Varma R. S., Zboril R. (2024). Chem. Rev..

[cit9] Kim J. H., Shin D., Kim J., Lim J. S., Paidi V. K., Shin T. J., Jeong H. Y., Lee K.-S., Kim H., Joo S. H. (2021). Angew. Chem., Int. Ed..

[cit10] Cho J., Lim T., Kim H., Meng L., Kim J., Lee S., Lee J. H., Jung G. Y., Lee K.-S., Viñes F., Illas F., Exner K. S., Joo S. H., Choi C. H. (2023). Nat. Commun..

[cit11] Muravev V., Spezzati G., Su Y.-Q., Parastaev A., Chiang F.-K., Longo A., Escudero C., Kosinov N., Hensen E. J. M. (2021). Nat. Catal..

[cit12] Zhang Z., Tian J., Lu Y., Yang S., Jiang D., Huang W., Li Y., Hong J., Hoffman A. S., Bare S. R., Engelhard M. H., Datye A. K., Wang Y. (2023). Nat. Commun..

[cit13] Qiao B., Wang A., Yang X., Allard L. F., Jiang Z., Cui Y., Liu J., Li J., Zhang T. (2011). Nat. Chem..

[cit14] Han B., Guo Y., Huang Y., Xi W., Xu J., Luo J., Qi H., Ren Y., Liu X., Qiao B., Zhang T. (2020). Angew. Chem., Int. Ed..

[cit15] Strayer M. E., Binz J. M., Tanase M., Kamali Shahri S. M., Sharma R., Rioux R. M., Mallouk T. E. (2014). J. Am. Chem. Soc..

[cit16] Jones J., Xiong H., DeLaRiva A. T., Peterson E. J., Pham H., Challa S. R., Qi G., Oh S., Wiebenga M. H., Hernández X. I. P., Wang Y., Datye A. K. (2016). Science.

[cit17] Kim J., Kim W., Seo Y., Kim J.-C., Ryoo R. (2013). J. Catal..

[cit18] Kuhn J. N., Tsung C.-K., Huang W., Somorjai G. A. (2009). J. Catal..

[cit19] Rodrigues A. C. C., Fontes Monteiro J. L. (2006). J. Therm. Anal. Calorim..

[cit20] de Araujo L. R. R., Schmal M. (2000). Appl. Catal., A.

[cit21] Ryoo R., Cho S. J., Pak C., Kim J. G., Ihm S. K., Lee J. Y. (1992). J. Am. Chem. Soc..

[cit22] Ryoo R., Cho S. J. (1993). Stud. Surf. Sci. Catal..

[cit23] Munoz-Paez A., Koningsberger D. C. (1995). J. Phys. Chem..

[cit24] Kwak J. H., Hu J., Mei D., Yi C.-W., Kim D. H., Peden C. H. F., Allard L. F., Szanyi J. (2009). Science.

[cit25] Yuan Y., Huang E., Hwang S., Liu P., Chen J. G. (2024). Nat. Commun..

[cit26] Plessow P. N., Abild-Pedersen F. (2016). ACS Catal..

[cit27] Park H., Park H., Kim J.-C., Choi M., Park J. Y., Ryoo R. (2021). J. Catal..

