# Supplementary material for: Silica-supported Pt(0) single-atom catalyst chemisorbing hydrogen but converting to Pt(iv) by ambient oxygen
Source: RSC Adv. 2026 Mar 2;16(13):11557–61. doi: 10.1039/d6ra00018e (PMC12951402; doi:10.1039/d6ra00018e)
Supplement: RA-016-D6RA00018E-s001 [file RA-016-D6RA00018E-s001.pdf]

Supplementary Information (SI) for RSC Advances.
This journal is © The Royal Society of Chemistry 2026

Electronic Supplementary Information (ESI)

Silica-supported Pt(0) single atom catalyst chemisorbing hydrogen but converting to Pt(IV) by ambient oxygen

Gwang-Jin Na, *Jongha Hwang*, *and Ryong Ryoo**

Department of Energy Engineering / Korea Institute of Energy Technology (KENTECH), Naju, Jeonnam 58330, Korea

*Corresponding author:
E-mail: rryoo@kentech.ac.kr
Orcid: 0000-0003-0047-3329

Experimental sections

H_2 temperature-programmed reduction.
The H_2-TPR profile (**Fig. S1**) was obtained manually by using a laboratory-built glass volumetric apparatus. 500 mg of 0.02 wt% Pt/SiO_2 was placed in a Pyrex reactor and O_2-treated at 300 °C prior to H_2-treatment. After cooling to room temperature, the sample was evacuated under an ultrahigh vacuum for 30 min to ensure complete degassing. The reactor was then immersed in a thermostatic bath maintained at 25 °C. Subsequently, H_2 gas was dosed into the apparatus to a pressure of approximately 13 kPa and allowed to diffuse into the reactor. The reactor was heated to the target temperature (ramping rate, 2.5 °C min^{-1}) and held for 30 min to complete reduction of Pt. After the reduction at each temperature, the sample was cooled again to 25 °C, and the decrease in gas pressure was monitored relative to the initial H_2 pressure. The amount of H_2 uptake was calculated in the same manner as obtained from hydrogen chemisorption measurements.

Preparation of supported metal catalysts.
High-purity grade silica gel (Davisil Grade 633, BET surface area of 480 m^2 g^{-1}) was purchased from Sigma-Aldrich. A siliceous MFI zeolite with a mesopore/micropore hierarchy was synthesized in a high-purity form, using a diammonium surfactant-type dual structure-directing agent [27]. These silicas were used after dehydration in air at 400 °C. $Pt(NH_3)_4(NO_3)_2$ (99.995%, Sigma-Aldrich) was employed as a Pt precursor. The preparation of Pt/SiO_2 was carried out *via* incipient-wetness impregnation [6].
For the preparation of 0.02 wt% Pt/SiO_2, 0.375 mL of distilled water corresponding to the silica pore volume was prepared so as to contain 0.20 mg of $Pt(NH_3)_4(NO_3)_2$. The solution was added dropwise onto 500 mg of SiO_2 powder inside a vial while the mixture was thoroughly rubbed with a spatula. After the lid of the vial was closed, the vial was maintained overnight at 60 °C in a drying oven, for homogeneous distribution of the Pt precursor. Afterwards, the lid was opened for overnight inside the oven at 60 °C. The sample powder, dried in this manner, was transferred into a borosilicate glass, U-shaped reactor fitted with two fritted glass disks. The sample in the reactor was gradually heated to 300 °C under a high flow of O_2 (ramping rate of 0.5 °C min^{-1}, flow rate of 1,000 cm^3 min^{-1} g_{cat}^{-1}) and maintained there for 2 h. After the reactor was cooled to room temperature, the O_2 gas was removed by He or N_2 purging, or by evacuation. Subsequently, the sample in the reactor was treated with a flow of H_2 while the temperature was increased to 200 °C (ramping rate: 2.5 °C min^{-1}, flow rate of 400 cm^3 min^{-1} g_{cat}^{-1}) and maintained there for 2 h. Afterwards, the Pt/SiO_2 sample was put under high vacuum for 2 h at 200 °C, in order to desorb hydrogen before chemisorption, or other measurements.
When there were no *in-situ* measurements, the H_2-treated Pt/SiO_2 sample was shortly put under vacuum at 200 °C. Then, after cooling to room temperature, the sample was passivated in a flow of 0.1 vol% O_2/N_2. The passivated Pt/SiO_2 sample was stored in a desiccator and re-treated with H_2 at 200 °C for chemisorption and other measurements like a freshly prepared sample.

Hydrogen chemisorption measurements.
A volumetric measurement apparatus, equipped with a Baratron capacitance manometer, was built with borosilicate glass. For accurate measurements of gas adsorption, the volume changes caused by the diaphragm deflection in the manometer were taken into account as follows: $V = V_0 + kP$, where V is the corrected volume according to pressure P, V_0 is the volume at zero pressure, and k is assumed to be a constant. The values of V_0 and k were determined from a V-*vs.*-P plot, which was obtained by helium gas expansion from a known-volume flask to the gas-dosing volume, repeating at various initial pressures (see **Fig. S12**).
Volumetric measurement of H_2 gas adsorption was carried out at 25.0 °C, maintaining the sample cell containing 0.5 g of 0.02 wt% Pt/SiO_2 in a constant-temperature bath. The adsorption isotherm over 4 – 25 kPa was extrapolated to zero pressure. The extrapolated value, expressed in terms of H/Pt, is referred to as the total hydrogen chemisorption. The detecting limit for 0.02 wt% Pt/SiO_2 was 0.1 H/Pt when the diaphragm

deflection effect was calibrated in this manner. For samples with higher Pt loadings, smaller amounts of samples could be used to achieve sufficiently high accuracy. However, when the diaphragm-deflection effect was not considered, conventional method extrapolating adsorption isotherms was difficult to apply for samples with less than 0.5 wt% Pt.

STEM and XANES measurements.

Atomic resolution HAADF-STEM images were obtained with passivated Pt/SiO_2 samples. The STEM analysis was performed at 300 kV using Spectra Ultra microscopes (Thermo Fischer Scientific) equipped with double Cs correctors, at KENTECH Shared Research Facility, and KAIST Analysis Center for Research Advancement. HAADF-STEM images were taken through thin edges of silica particles supported with Pt, which were placed onto a carbon TEM grid (Holey Carbon-Au, 200 mesh, 100 micron) by dropping an ethanol suspension of particles to the grid and letting it dry.

XAFS was measured at the Pt L_3 edge at Pohang Accelerator Laboratory (10C-Wide XAFS beamline). A transmission mode was used for samples with high Pt content while fluorescence detection was chosen for 0.02 wt% Pt/SiO_2 using a silicon drift detector (RaySpec Sirius SD). For XAFS investigation of the O_2-treated state, the Pt precursor-impregnated silica sample was heated in the powder state under a flow of O_2 at 300 °C. The treated sample was open to air, and pressed into a round-shaped pellet (diameter, 10 mm). This pellet was stored in a desiccator to minimize moisture absorption until the XAFS measurements under ambient atmospheric conditions. In the case of the H_2-treated state, the sample pellet after the O_2 treatment was placed inside a borosilicate glass tube reactor, which was connected to a Kapton-windowed XAFS cell. After heating at 200 °C under a flow of H_2, the Pt/SiO_2 pellet was internally transferred into the XAFS cell by tilting. The XAFS data including XAENS and EXAFS were collected under H_2 therein. All data were calibrated with Pt foil using ATHENA and ARTEMIS software. In the XANES linear combination fitting, the spectra of Pt foil and PtO_2 were used as references for Pt^0 and Pt^{4+}, respectively.

Catalytic reaction measurements.

The O_2-treated Pt/SiO_2 powder was gently pressed, crushed, and sieved to collect soft pellets of 40 – 60 mesh. 20 mg of the sieved pellets were loaded into a borosilicate glass reactor (inner diameter, 8 mm). The Pt/SiO_2 sample was treated inside the reactor with a flow of H_2 under heating at 200 or 400 °C (2 h to reach, and maintained there for 2 h). After this treatment, the sample temperature was lowered to –20 °C under a flow of N_2 gas, which was cooled through a refrigerated thermostatic circulator. The N_2 flow was then switched to a flow of gas at –20 °C consisting of 0.60 vol% n-C_6 in N_2 (flow rate of 5 cm^3 min^{-1}). This gas mixture, which was prepared by bubbling N_2 through liquid n-C_6 at –40 °C, was continuously fed for 0.5 h until the catalyst bed became fully equilibrated with n-C_6. Afterwards, the n-C_6/N_2 flow (5 cm^3 min^{-1}) was added with a flow of H_2 (5 cm^3 min^{-1}) to start the hydrogenation reaction. The H_2/n-C_6 molar ratio was 164, with the n-C_6 weight hourly space velocity (WHSV) of 1,700 h^{-1} (based on the total weight of catalyst including the support).

The hydrogenation of C_3 and C_2 olefins was performed using a premixed gas cylinder consisting of 1 vol% olefin balanced with He. The reaction was conducted at 0 °C using 20 mg of catalyst, with a WHSV of 1,400 h^{-1} for C_3, and 940 h^{-1} for C_2, while maintaining the H_2/C_3 and H_2/C_2 molar ratios to be 100.

Reaction products were analyzed by an on-line gas chromatography instrument equipped with a flame ionization detector (FID). An HP-5ms column was used to separate the product from reactant in the case of n-C_6 hydrogenation. A GS-GasPro column was used in the cases of C_3 and C_2 hydrogenation. The olefin conversion was calculated on a carbon basis, without coke deposition.

Supplementary Figures

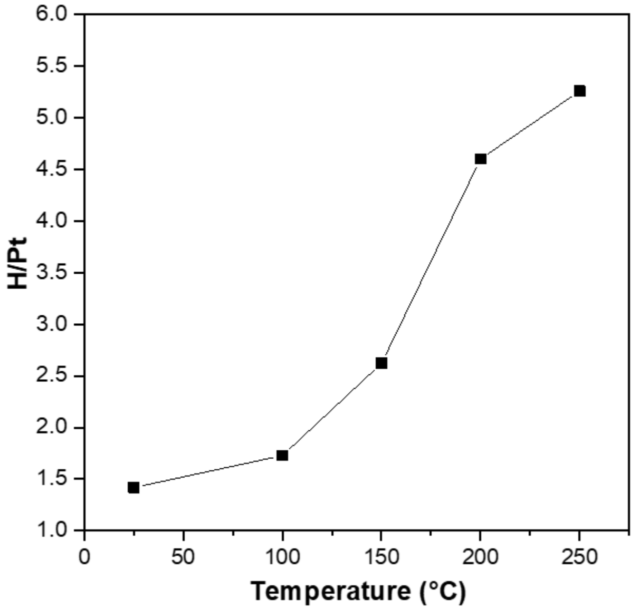

Fig. S1. Evolution of the H/Pt ratio during the TPR run of a 0.02 wt% Pt/SiO_2 sample upon temperature increase under 6.8 kPa of H_2. Prior to the uptake measurement, the Pt/SiO_2 sample was prepared by impregnation of $Pt(NH_3)_4(NO_3)_2$, and a subsequent treatment with O_2 at 300 °C. Each point was determined from a pressure decrease. The hydrogen uptake was due to the conversion of PtO_2 to Pt(0) for 0.5 h at a given temperature, and subsequent hydrogen chemisorption for 0.5 h at 25 °C.

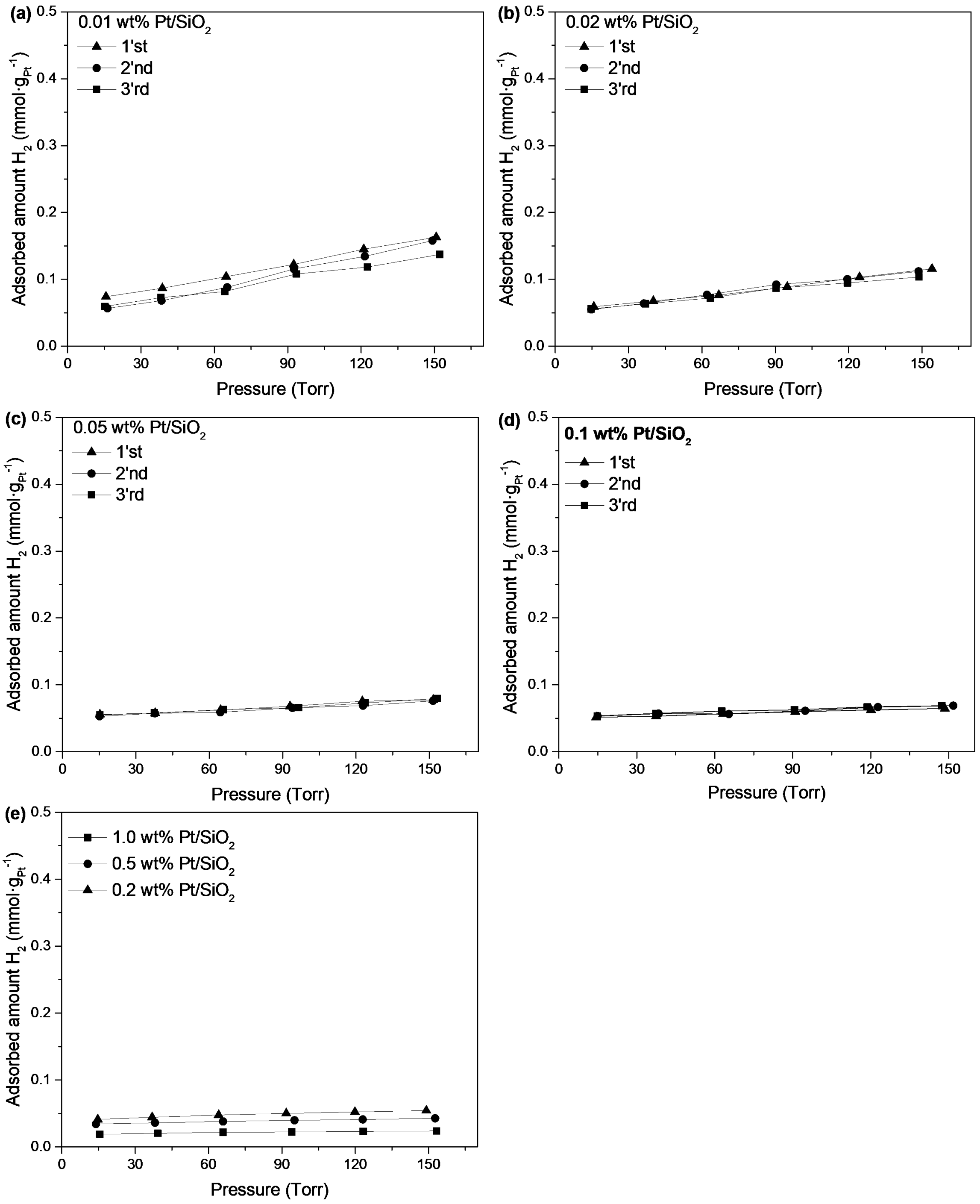

Fig. S2. Hydrogen adsorption isotherms of Pt/SiO_2 samples with different Pt loadings: (a) 0.01 wt%, (b) 0.02 wt%, (c) 0.05 wt%, (d) 0.1 wt%, and (e) 0.2 – 1.0 wt%.

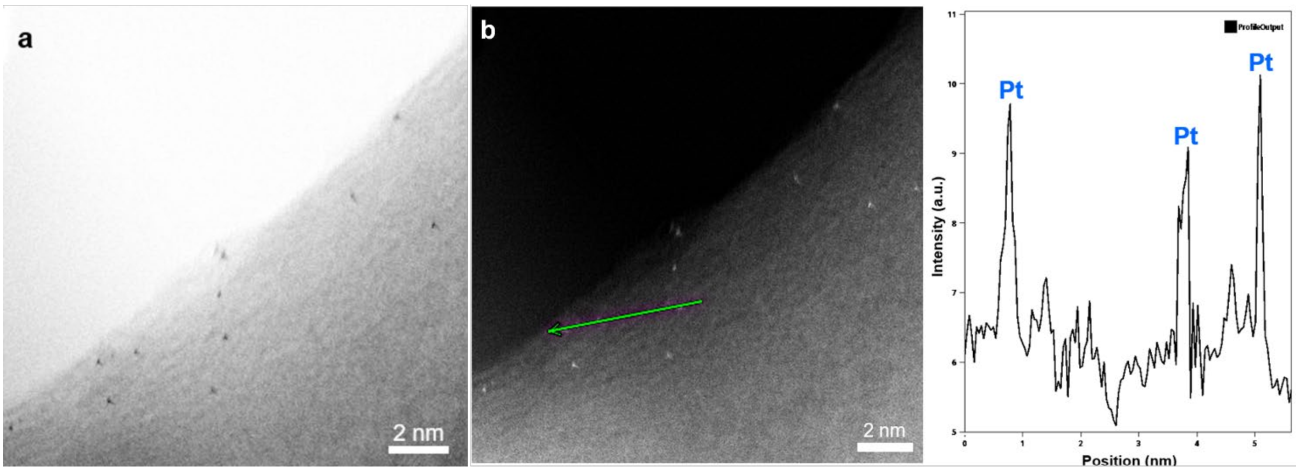

Fig. S3. (a) A high-contrast HAADF-STEM image of 0.02 wt% Pt/SiO_2 and (b) the corresponding line-scan results

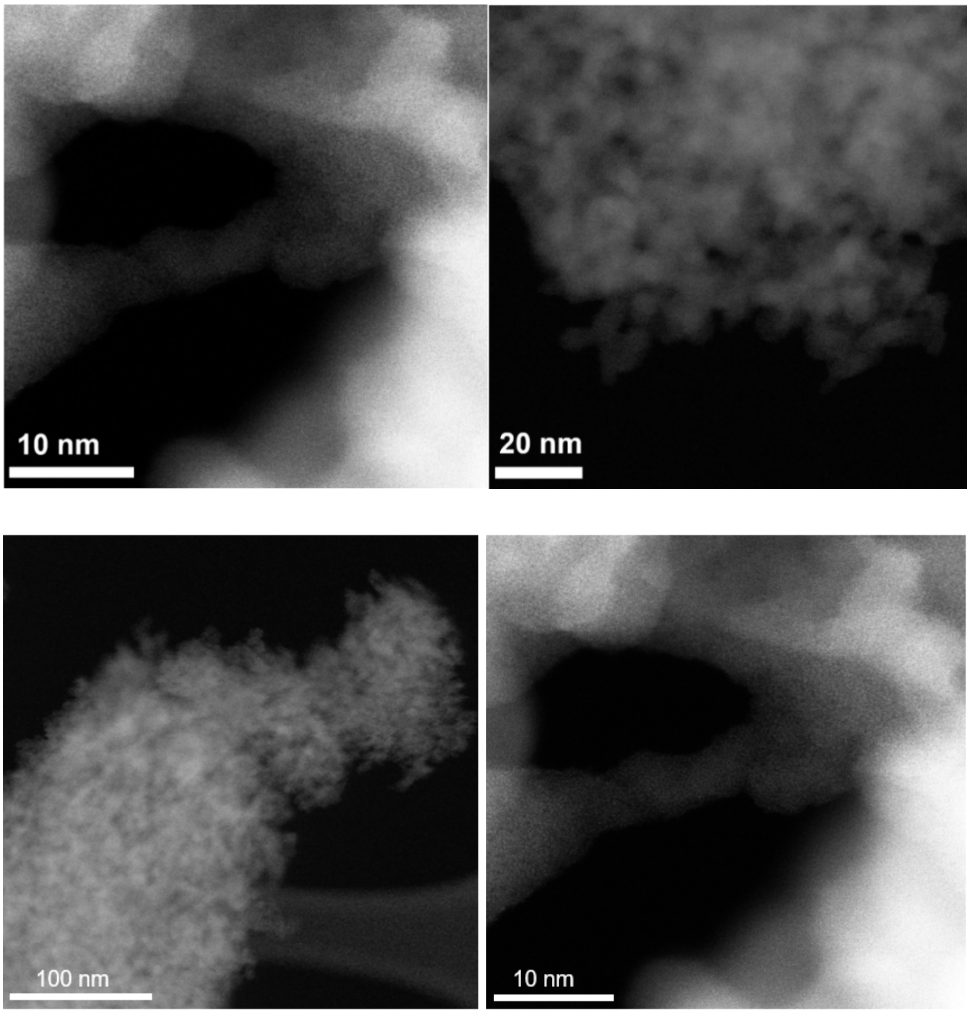

Fig. S4. HAADF-STEM images of 0.02 wt% Pt/SiO_2 after H_2 treatment at 200 °C with a relatively low magnification.

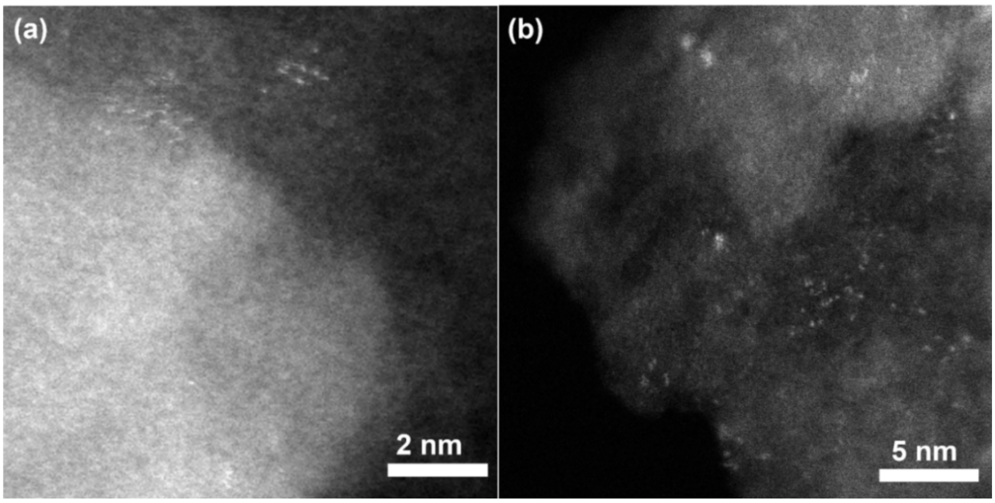

Fig. S5. HAADF-STEM images of Pt/SiO_2 with Pt loadings of 0.05 wt% (a) and 0.1 wt% (b) after H_2 treatment at 200 °C.

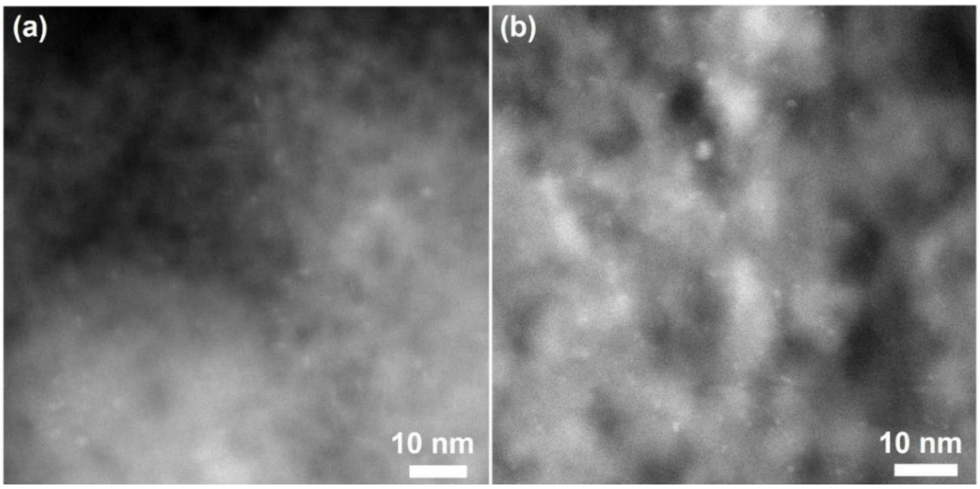

Fig. S6. HAADF-STEM images of 0.02 wt% Pt/SiO_2 after H_2-treatment at (a) 300 °C and (b) 400 °C.

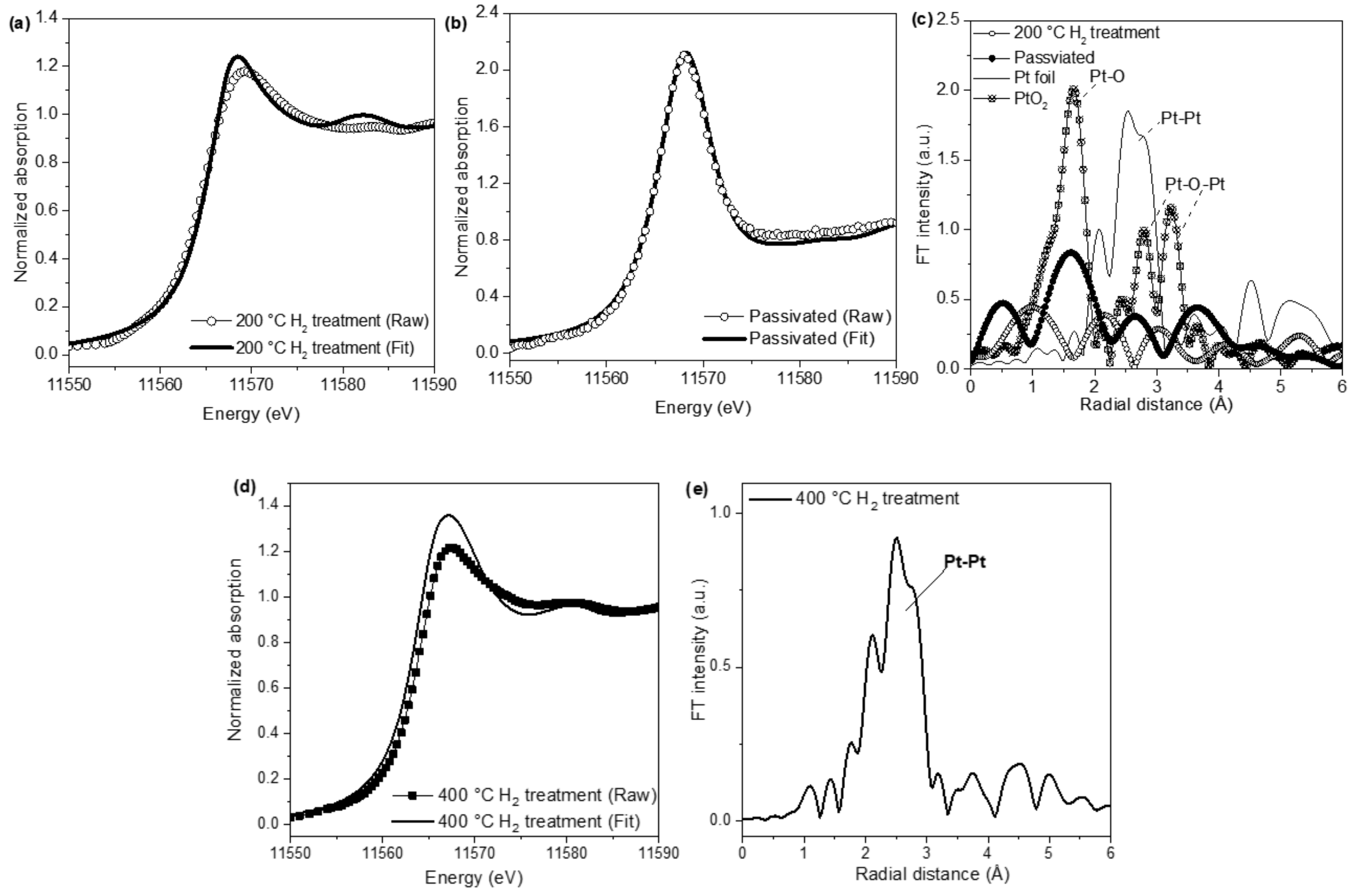

Fig. S7. Pt L_3 edge XANES linear combination fitting results for 0.02 wt% Pt/SiO_2 after 200 °C H_2 treatment (a) and passivation with 0.1% O_2/N_2 (b). Fourier transformed EXAFS data of 0.02 wt% Pt/SiO_2 after 200 °C H_2 and after subsequent passivation. Pt L_3 edge XANES linear combination fitting results for 0.02 wt% Pt/SiO_2 after H_2 treatment at 400 °C (d), together with the corresponding Fourier transformed EXAFS data (e), showing the formation of Pt nanoparticles.

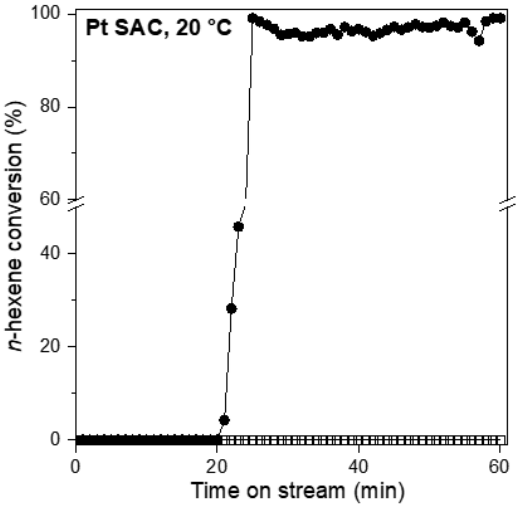

Fig. S8. Hydrogenation of *n*-C_6 olefin over 0.02 wt% Pt/SiO_2 in the case of single-atom Pt prepared by 200 °C H_2 treatment. (a) (□): reaction at 20 °C exhibiting no catalytic activity. (●): reaction at 20 °C exhibiting no catalytic activity for the first 20 min, after which the conversion increased to nearly 100%. (Reaction conditions: 20 mg catalyst, H_2/*n*-C_6 = 164, WHSV = 1,700 h^{-1}).

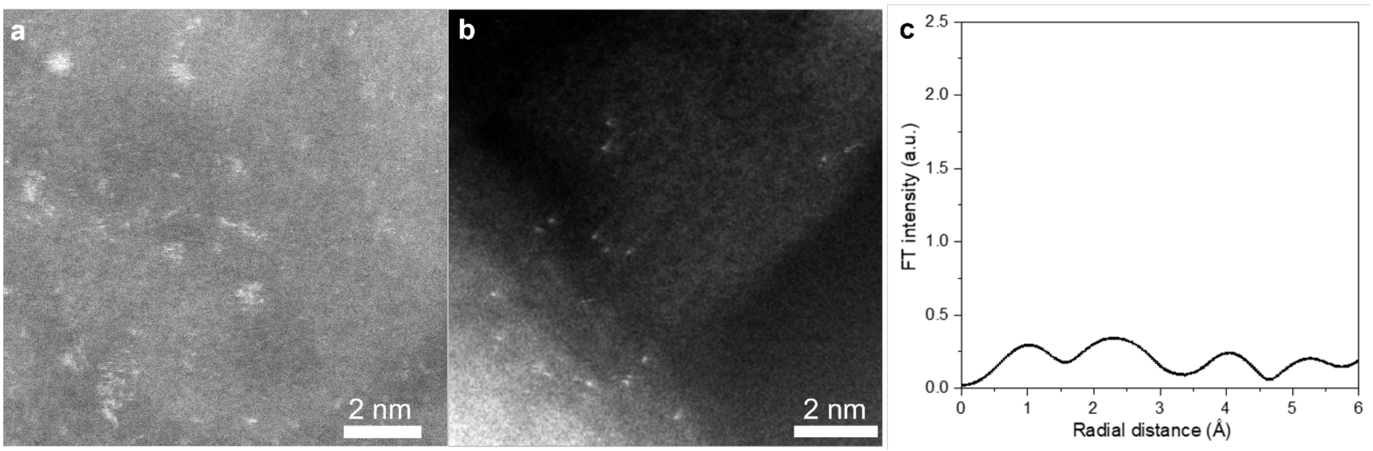

Fig. S9. HAADF-STEM images of 200 °C H_2-treated 0.02 wt% Pt/SiO_2 recovered after *n*-C_6 hydrogenation: (a) after and (b) without thermal runaway. (c) Fourier transformed EXAFS data of 0.02 wt% Pt/SiO_2 after *n*-C_6 hydrogenation without thermal runaway.

Table S1. Olefin hydrogenation turnover rates on 0.02 wt% Pt/SiO_2, exhibiting a dramatic difference between single atomically dispersed Pt catalyst (SAC) and atom-agglomerated clusters.

Dispersed state of Pt	Olefins	T(°C) [a)]	Olefin feeding (C_n/Pt/s) [b)]	Turnover rate (C_n/Pt/s) [c)]
SAC	*n*-C_6	–20	1.1	N/D [d)]
	C_3		1.8	
	C_2		1.8	
Cluster	*n*-C_6	0	1.1	2.5 x10^{-2}
	C_3		1.8	1.7 x10^{-2}
	C_2		1.8	1.8 x10^{-2}

[a)] Reaction temp. [b)] Number of C_n olefin molecules fed per total Pt atom per second. [c)] Number of C_n molecules converted per total Pt atom per second. [d)] Non-detectable below 1.0×10^{-4}.

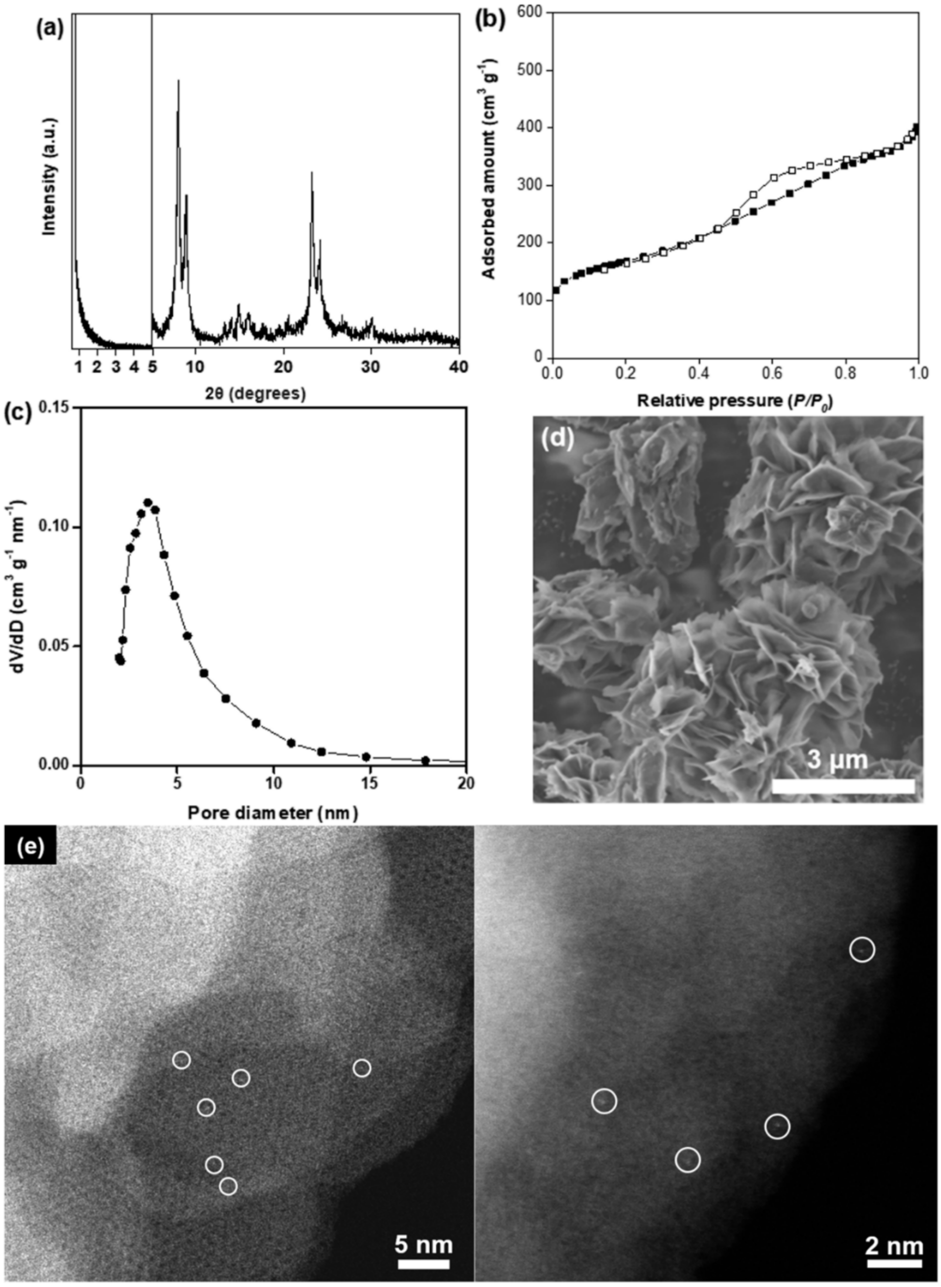

Fig. S10. Structural characterization of lab-made high-purity silica in the form of hierarchically meso-microporous MFI zeolite: (a) XRD patterns, (b) N_2 adsorption–desorption isotherms, (c) pore size distributions, and (d) SEM and (e) HAADF-STEM images of 0.02 wt% Pt/SiO_2 prepared using the high-purity silica. Pt single atoms, indicated by white circles, are dispersed at dimensions smaller than the micropores of the zeolite framework.

Table S2. ICP-OES results for impurity analysis.

Sample	Na (mg/kg)	K (mg/kg)	Pt (mg/kg)
Davisil silica [1)]	389.3	N/D [2)]	N/D
Lab-made silica	80.2	N/D	N/D

[1)] Davisil Grade 633, high-purity grade of silica gel obtained from Sigma-Aldrich. [2)] N/D: Not detected. Data were obtained using an iCAP7400DUO instrument (Thermo Fischer).

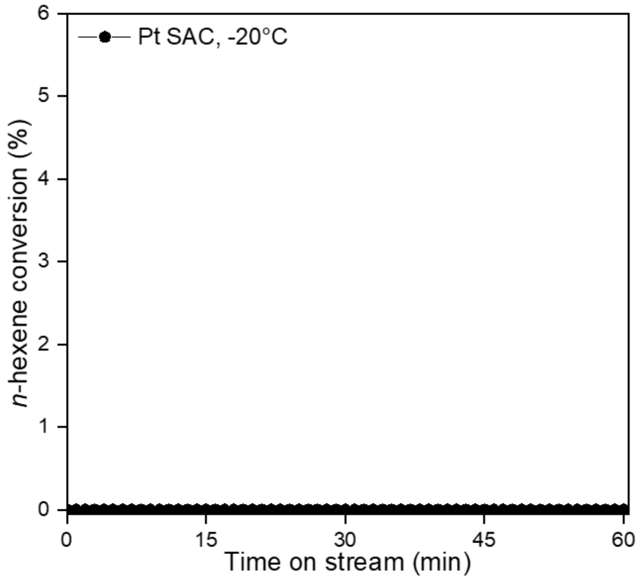

Fig. S11. Hydrogenation of *n*-C_6 olefin on 0.02 wt% Pt/SiO_2 using a high-purity SiO_2 which was synthesized in the form of hierarchically meso-microporous MFI zeolite. Reaction at –20 °C exhibiting no catalytic activity, on the single-atom Pt catalyst prepared through 200 °C H_2 treatment. (Reaction conditions: 20 mg catalyst, H_2/*n*-C_6=164, WHSV= 1,700 h^{-1})

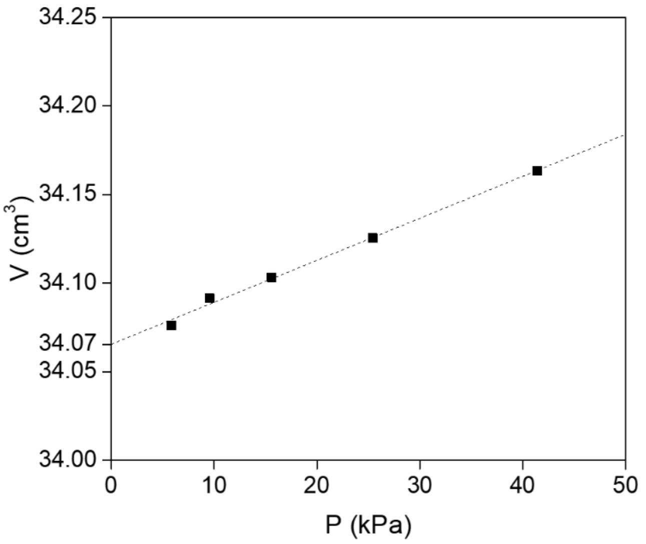

Fig. S12. A V-*vs.*-P plot obtained by helium gas expansion from a flask of 54.202 cm^3 to the gas-dosing volume (V), starting at various initial pressures. P indicates the final pressures.
